# Dexmedetomidine and Netrin-1 Combination Therapy Inhibits Endoplasmic Reticulum Stress by Regulating the ERK5/MEF2A Pathway to Attenuate Cerebral Ischemia Injury

**DOI:** 10.3389/fnins.2021.641345

**Published:** 2021-01-28

**Authors:** Jiang-Wen Yin, Jia Li, Yi-Min Ren, Yan Li, Rui-Xue Wang, Sheng Wang, Yun-Xia Zuo

**Affiliations:** ^1^Department of Anesthesiology, West China Hospital of Sichuan University, Chengdu, China; ^2^Laboratory of Anesthesia and Critical Care Medicine, Translational Neuroscience Center, West China Hospital of Sichuan University, Chengdu, China; ^3^Department of Anesthesiology, First Affiliated Hospital, School of Medicine, Shihezi University, Shihezi, China; ^4^Department of Anesthesiology, The Affiliated Hospital of Guizhou Medical University, Guiyang, China; ^5^Department of Anesthesiology, First Affiliated Hospital of USTC, Division of Life Sciences and Medicine, University of Science and Technology of China, Hefei, China

**Keywords:** dexmedetomidine, Netrin-1, endoplasmic reticulum stress, cerebral ischemia/reperfusion, oxygen-glucose deprivation, apoptosis

## Abstract

The complexity of hard-to-treat diseases such as ischemic stroke strongly undermines the therapeutic potential of available treatment options. Therefore, current developments have gently shifted from a focus on monotherapy to combined or multiple therapies. Both dexmedetomidine and Netrin-1 have anti-neuronal apoptosis effects, but the mechanism is still unclear. The study aimed to estimate the efficacy of dexmedetomidine and Netrin-1 combination therapy against ERS-induced apoptosis after cerebral ischemia injury *in vivo* and *in vitro*, and whether the mechanism is related to the ERK5/MEF2A pathway. Adult male Sprague-Dawley rats were subjected to middle cerebral artery occlusion (MCAO) *in vivo*, 90 min ischemia and 24 h reperfusion. The hippocampus slices used to establish oxygen-glucose deprivation (OGD) injury model *in vitro*. Neterin-1 and Dexmedetomidine were pretreated and post-treated, respectively, before and after the model establishment. MEF2A knockdown was performed by microinjection of AAV9-MEF2A RNAi vector. Orthodromic population spike (OPS) at the end of reoxygenation were recorded. Neurobehavioral tests, TTC staining, Nissl staining, TUNEL staining were performed to assess the effect of the drugs. The expression of CHOP, GRP78, MEF2A, ERK5, and p-ERK5 were investigated by Western blot and immunofluorescence staining. Neurological deficit score, infarct volume, the expression of GRP78, CHOP, and neural apoptotic rate of MCAO group increased markedly. Combination of dexmedetomidine and Netrin-1 resulted in lower infarct volumes and fewer neurological impairments, higher OPS recovery rate, and less damaged and apoptotic cells after cerebral ischemia injury. Furthermore, expression levels of GRP78 and CHOP decreased in the combination therapy group, and it was more effective than the single drug group. Meanwhile, Combination of dexmedetomidine and Netrin-1 increased MEF2A expression and promoted ERK5 phosphorylation. However, the protective effect of dexmedetomidine combined with Netrin-1 in improving neurological function was significantly eliminated by pre-knockdown MEF2A. The neuroprotective effects of dexmedetomidine combined with Netrin on cerebral ischemia-reperfusion injury and hippocampal hypoxia injury in terms of ERS. The synergistic effect of combination therapy is related to the activation of ERK5/MEF2A signaling pathway.

## Introduction

Ischemic stroke is still the leading cause of disability and death in the world (Stinear et al., 2020). The best treatment is to open the occluded vessels as soon as possible and rescue the ischemic penumbra in time (Meyers et al., 2019). However, the recovery of cerebral blood flow can lead to cerebral ischemia-reperfusion injury, aggravate neuronal apoptosis, lead to nerve injury cascade reaction, and affect the recovery of nerve function and prognosis (Broughton et al., 2009). Therefore, how to reduce cerebral ischemia-reperfusion injury and protect nerve function while improving cerebral perfusion has attracted more attention. The complexity of hard-to-treat diseases such as ischemic stroke strongly undermines the therapeutic potential of available treatment options. Therefore, current developments have gently shifted from a focus on monotherapy to combined or multiple therapies since the synergy of therapeutic agents or techniques give rise to ostentatious super additive (namely “1 + 1 > 2”) therapeutic effects (Poustchi et al., 2020).

Endoplasmic reticulum (ER) is the leading site of protein processing and transportation in and out of neurons. Calcium depletion, ischemia, and hypoxia, glucose, and nutrient deficiency can lead to the aggregation of unfolded or misfolded proteins in ER, which leads to ER stress (Marchi et al., 2018). In response to endoplasmic reticulum stress, cells form a self-protection signal transduction pathway called unfolded protein response (UPR) (Jiwon and Qi, 2018). If severe and persistent endoplasmic reticulum stress was induced by cerebral ischemia, the unfolded protein could not maintain the ER stability; then further initiate the apoptosis process, eventually lead to neuron death and affect the long-term neural function (Qiu et al., 2017).

At present, PERK, IRE1A, and ATF6 are known as upstream pathways of UPR (Jiwon and Qi, 2018). Molecular chaperone protein GRP78 can connect the binding domains of the three proteins and play an inhibitory role (Ouyang et al., 2012). The accumulation of misfolded proteins in the ER can activate the above processes. When mild ER stress occurs, UPR can maintain ER homeostasis by up-regulating ER chaperone molecules, thus playing a compensatory cytoprotective role. However, sustained or excessive ER stress can cause caspase-12 to enter the cytoplasm from endoplasmic reticulum, activate caspase-9, and activate caspase-3 finally, trigger ER stress-mediated apoptosis. The apoptotic process is initiated by the transcriptional induction of C/EBP homologous protein (CHOP) (Chen et al., 2020). Therefore, GRP78, and CHOP are commonly used as intracellular biomarkers for ER stress.

The effect of transcription factors activity on cell apoptosis is the hot spot of the current research. MEF2A, a subtype of nuclear element in myogenic cells, was up-regulated in the cerebellum and hippocampus (Wu et al., 2017). Studies have shown that inhibition of MEF2A transcriptional activity can promote neuronal apoptosis, but its role and regulatory mechanism in cerebral ischemia-reperfusion injury model are still unclear. ERK5 is an essential contributor to cell survival. Our previous research found that extracellular regulated protein kinase 5 (ERK5) can reduce ER stress (Nam et al., 2017). Up-regulation of *p*-ERK5 play a crucial role in reducing neuronal apoptosis in the OGD model. Besides, MEF2A is a specific substrate for ERK5. The C-terminal of ERK5 includes a transcriptional activation region (aa 664–789) and a MEF2 interaction region (aa 440–501), which are essential for the synergistic activation of MEF2 (Katsarou et al., 2011; Lee et al., 2011). Therefore, we speculated that ERK5/MEF2A signaling pathway may be involved in the ER stress pathway and apoptosis process of cerebral ischemia-reperfusion injury.

As a highly selective central sedative, dexmedetomidine has been widely used for sedation in perioperative and severe patients (Su et al., 2016; Turan et al., 2020). Besides sedative and anti-sympathetic effects, dexmedetomidine can reduce the synthesis of free radicals, reduce the apoptosis of nerve cells, and reduce brain injury, ischemia-reperfusion injury, and glucose oxygen deprivation injury (Zhai et al., 2019; Zhu et al., 2019). Similarly, Netrin-1, a Netrins family representative, provides migration signals during the central nervous system’s growth and development. As a soluble secretory protein, it has high expression in the central nervous system’s development in many organisms (Ahn et al., 2020). It participates in the structural and functional reconstruction of the nervous system after injury (Terzi et al., 2020). To minimize ischemia-reperfusion injury, effective drugs, and combined applications are urgently needed. The purpose of this study was to demonstrate the neuroprotective effects of dexmedetomidine combined with Netrin on cerebral ischemia-reperfusion injury and hippocampal hypoxia injury in terms of ERS, apoptosis, and electrophysiology, whether the combination therapy is related to the activation of ERK5/MEF2A signaling pathway.

## Materials and Methods

### Ethics Statement

All animal experiments were conducted in accordance with the National Institute of Health Guide for the Care and Use of Laboratory Animals. The experimental procedures were approved by the Animal Research Committee by Shihezi University (protocol A2018-025-01) and West China Hospital of Sichuan University (protocol 2018159A).

### Animals and Grouping

Male Sprague-Dawley rats aged between 6 and 8 weeks old (180 ∼ 240 g) were used in this study. Rats were housed in the vivarium managed by the Division of Animal Lab Center of Shihezi University with a 12-h light/dark cycle and *ad libitum* access to food and water. Rats were anesthetized with an intramuscular injection (0.1 ml/100 g body weight) of anesthetics containing ketamine (60 mg/ml) and xylazine (10 mg/ml) for middle cerebral artery occlusion (MCAO), sham operation, or decapitation.

*In vivo*, rats were randomly assigned to five groups: sham group, MCAO group, Dexmedetomidine processing ischemia-reperfusion group (Dex group), Netrin-1 processing ischemia-reperfusion group (NT-1 group), and Dexmedetomidine combined with Netrin-1 treatment group (Dex + NT-1 group).

*In vitro* experiments with hippocampal slices, rats were randomly assigned to five groups: Normal group, Oxygen-Glucose deprivation group (OGD group), Dexmedetomidine processing OGD group (Dex group), Netrin-1 processing OGD group (NT-1 group), and Dexmedetomidine combined with Netrin-1 treatment group (Dex + NT-1 group).

### Protocol of Middle Cerebral Artery Occlusion (MCAO) Model

**FIGURE 1:**
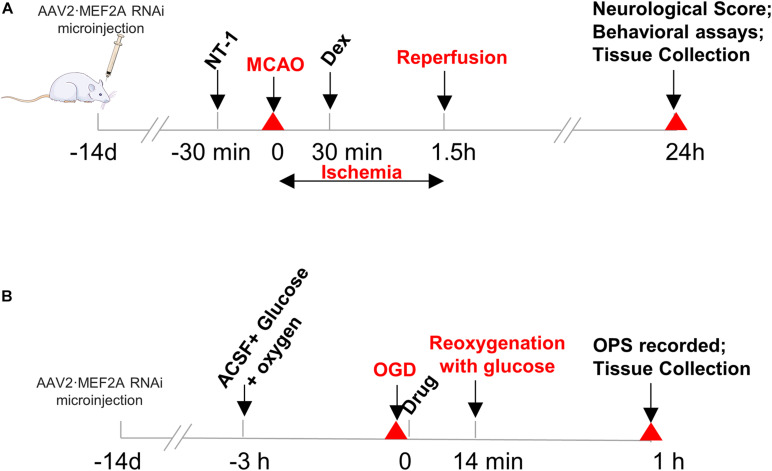
Protocols of overall experiments: **(A)** *in vivo* and **(B)** *in vitro*.

Focal cerebral ischemia was induced using the MCAO model as described previously (Peng et al., 2019). Briefly, the right common carotid artery (CCA), external carotid artery (ECA), and internal carotid artery (ICA) were exposed through a midline neck incision and carefully isolated from the surrounding tissues. The ECA was cut proximal to the lingual and maxillary artery branches. Other ECA branches were coagulated and transected. The ICA and vagus nerves were carefully isolated to avoid neurologic damage. A 3-0 monofilament nylon suture with a rounded tip was inserted into the ICA through the ECA stump until faint resistance was felt. The distance between the bifurcation of the CCA and the suture tip was 18.5 ± 0.5 mm. The skin was properly sutured, and the thread was retained by 1 cm to occlude the middle cerebral artery for 90 min, and rats were allowed to recover. Then, the thread was withdrawn to allow blood flow into the MCA. Sham-operated mice underwent the same procedure without thread insertion. In Dexmedetomidine group, 9 μg/kg of dexmedetomidine was infused into the tail vein at the onset of reperfusion for 30 min through micro-infusion pump. Rats in Netrin-1 group were injected 500 ng rNetrin-1 (50 ug/ml, R&D Systems, United States, #6419-N1-025) at 30 min prior to MCAO through the lateral ventricle of the brain stereo locator for a total of 10 μl for 10 min. Rats in Dex + NT-1 group first received Netrin-1 at 30 min prior to MCAO and were infused with dexmedetomidine at the onset of reperfusion for 30 min (Figure 1A).

### Protocol of Oxygen-Glucose Deprivation Model Orthodromic Population Spike (OPS) Test

Oxygen-glucose deprivation and test of OPS were conducted, as our research team described previously (Wang et al., 2010; Wang et al., 2016). In brief, hippocampal slices were incubated in ACSF solution (in mM):124 NaCl, 1.25 NaH_2_PO_4_, 3.6 KCl, 2.2 MgSO_4_, 26 NaOH, 2.2 CaCl_2_, and 10 glucose, and pre-perfused with 95% O_2_/5% CO_2_ at 34°C with pH 7.35 ∼ 7.45 for 3 h. Then, irrigation solution was replaced with glucose-free 95% N_2_/5%CO_2_-mixed ACSF to initiate the OGD. After 14 min of OGD, reoxygenation was performed by using 95% O_2_/5% CO_2_-mixed ACSF with glucose for 60 min (Figure 1B). After reoxygenation, slices were immersed in a constant irrigation tank at 34°C and continuously perfused with the same ACSF in 1.5 mL/min. Then, OPS was recorded. The stimulation electrode was placed in the Schaffer lateral branch pathway in CA3 region of the hippocampus, with the stimulus intensity of 0.6 mA. The recording pipettes (4 ∼ 10 MΩ) were pulled from borosilicate glass by using a Flaming-Brown horizontal puller (Model P2000, Sutter Instruments), was placed in the hippocampal pyramidal cell layer of CA1 area to record the OPS.

Hippocampal slices in OGD group without any drug treatment, Dex group in which culture medium was added with 10 μmol/L of dexmedetomidine (Kuhmonen et al., 2001), NT-1 group in which culture medium was added with 10 nmol/L of Netrin-1 (Yu et al., 2017), Dex + NT-1 group in which culture medium was added with 10 μmol/L of dexmedetomidine and 10 nmol/L of Netrin-1. During OGD and reoxygenation, slices were subjected to drug treatment, and the concentrations of all drugs were maintained. Abolish time, recovery time, recovery amplitude, and recovery rate of OPS were measured by electrophysiological system processing software. The OPS recovery rate was calculated by the percentage of brain slices in which the OPS amplitude recovery should reach 60% of primary OPS amplitude before OGD.

### AAVs Generation and AAVs Microinjections

SiRNA targets were designed based on MEF2A transcripts and primers were arranged for synthesis. The single-stranded primers were annealed into double-stranded Oligo sequences and connected to the double-enzymatic linear zed RNA interference vector (Hanbio Biotechnology Co., Ltd.). The KD efficiency was then evaluated by cotransfecting EGFP-tagged MEF2A with the MEF2A-shRNA vectors separately in HEK293 cells, and the KD efficiency was indicated by the reduction of the fluorescence signal expressed by the EGFP-MEF2A vector. The most effective sequence was chosen as follows: MEF2A-shRNA (GGGCAGTTATCTCAGGGTTCAA) and NC-siRNA (TTCTCCGAACGTGTCACGTAA). The selected oligos were then cloned into the linear zed pHBAAV-U6-MCS-CMV-EGFP vector (Hanbio Biotechnology Co., Ltd.) using T4 DNA ligase.

Viral injections were performed by using a stereotaxic apparatus (Zhongshi Dichuang Science and technology development Co., Ltd., China) to guide the placement of a Hamilton syringe fixed with beveled glass pipettes (Sutter Instrument, 1.0-mm outer diameter) into the hippocampus (The positioning coordinates are as follows: AP −3.3 mm; ML ± 2.3; DV −2.4 from the skull). A total of 1.0 μL of AAV2⋅MEF2A RNAi⋅GFP (1.1 × 10^12^ vg/mL), 1.0 μL of NC-RNAi (1.6 × 10^12^ vg/mL) was slowly injected into both sides of the hippocampus. Glass pipettes were left in place for at least 5 min. After injection, the rats were placed on a thermostatic heating pad for recovery and then returned to the home cage.

### Measurement of Cerebral Infarction Areas

After 24 h of reperfusion, animals were anesthetized again and sacrificed by decapitation. Brains were quickly isolated and sectioned into five coronal slices of 2 mm thickness. The slices were stained with 2% 2,3,5-triphenyle-tetrazolium chloride (TTC; Sigma-Aldrich, St. Louis, MO, United States) for 30 min at 37°C in the dark and fixed with 4% paraformaldehyde (PFA; Sigma-Aldrich) overnight.

The posterior surface of each slice was photographed under a digital camera and analyzed by Image J software (Rawak Software Inc., Stuttgart, Germany). The infarct volume (%) was calculated as the infarct area relative to the contralateral hemisphere area in each slice.

### Nissl Staining and Neuron Count

Animals were anesthetized at 24 h after reperfusion and transcardially perfused with ice-cold Ringer’s solution followed by 4% paraformaldehyde, then processed for paraffin embedding. Paraffin sections were deparaffinized in xylene and dehydrated in gradations of ethanol. The sections were stained with Cresyl violet (Solarbio, China) for 1 h at 50°C. The number of Nissl’s bodies in CA1 region of hippocampus was observed under microscope was quantified by an observer without knowledge of the experiment.

### TUNEL Staining

TUNEL assay was performed by using the *in Situ* Cell Death Detection Kit (Roche, Switzerland, Germany) following the manufacturer’s instructions. After TUNEL staining, sections were then incubated with DAPI-containing mounting media. The 10 × objective lens plus 1.5 × optical zoom Z-stack images were obtained using the Zeiss confocal microscope (LSM710). Then, the number of TUNEL^+^ and DAPI^+^ cells within the whole image views was quantified by using Image J software. The density (per 1 mm^2^) of TUNEL^+^ cells were calculated.

### Neurological Behavioral Assessment

Animals were quantitatively examined for neurological deficits by an observer who was blinded to the groups at 24 h after reperfusion using modified Neurological Severity Scores (mNSS) as previously reported (Cai et al., 2020). The mNSS mainly includes: Motor tests, Beam Balances test and Reflexes absent and abnormal movements. The sum of all points was used as the neurobehavioral deficit score.

### Step-Through Test

Step-through test is one of the most commonly used tools of cognitive function in experimental stroke studies (Komotar et al., 2007). As rats show a tendency to prefer darkness, we designed an apparatus divided by a gate into two parts: a dark compartment and a brightly lit compartment. The floor of the dark compartment was made of electrified copper grid and connected to a stimulator. When rats entered the dark room, an electrical shock (0.45 mA) was delivered to the grid in the dark compartment by the stimulator. Each rat was put in the brightly lit compartment, after 10 s, the gate was opened. The rat got the electric shock immediately when it entered the dark compartment. After 5 min, the rat was removed. The learning acquisition trial ended when the rat did not enter into the dark room within 2 min. At 24 h following MCAO injury, this procedure was repeated without the electric shock. We recorded the time taken (latency) for rats to enter into the dark room and number of errors within 5 min. Experimenters were blind to the groups.

### Immunofluorescence Staining

Immunofluorescence staining was performed as previously reported (Zhang et al., 2019). Frozen section of brain tissue were permeabilized with Triton X-100 (0.2%, 3 min). Blocking with 10% BSA for 1 h at 37°C. The following primary antibodies were used: rabbit anti-CHOP (1:100; ThermoFisher #PA5-104528), rabbit anti-GRP78 (1:200, Abcam #ab21685), rabbit anti-MEF2A (1:200, Abcam #ab76063), and rabbit anti-p-ERK5 (1:100, ThermoFisher #44-612G). The following Alexa conjugated secondary antibodies were used: goat anti-rabbit (Alexa Fluor^®^ 488, 1:500, Abcam #ab150077), goat anti-rabbit (Alexa Fluor^®^ 555, 1:500, Abcam #ab150078), and goat anti-rabbit (Alexa Fluor^®^ 594, 1:500, Abcam #ab150080). After nucleus labeling with DAPI or propidium iodide, sealed the tablet with an anti-fluorescence attenuation sealant. For quantification of fluorescent intensity, sections from all groups were stained and imaged with exactly the same protocol.

### Western Blotting Analysis

The proteins were separated from the infarcted hemisphere hippocampal tissue, and then separated by 10% sodium dodecyl sulfate polyacrylamide gel electrophoresis (SDS-PAGE) and then transferred to polyvinylidene fluoride (PVDF) membranes. Blocking by 10% skimmed milk or BSA for 2 h, and then the membranes were incubated at 4°C overnight with rabbit anti-CHOP (1:1,000; ThermoFisher #PA5-104528), rabbit anti-GRP78 (1:1,000, Abcam #ab21685), rabbit anti-MEF2A (1:1,000, Abcam #ab76063), and rabbit anti-p-ERK5 (1:1,000, ThermoFisher #44-612G), and β-actin (1:1,000, ZSGB-BIO, Beijing, China). After cleaning with TBST buffer, the membranes were incubated with secondary antibodies labeled by horseradish peroxidase (1:10,000, ZSGB-BIO, Beijing, China) for 2 h. Finally, chemiluminescent reagent (ThermoFisher, Waltham, MA, United States) were added to examine blots, and analyzed blots quantitatively by Image J software.

### Statistics

All statistical analyses were performed using Graphpad prism 8.0 (GraphPad Software, La Jolla, CA, United States). Normality test was applied before one-way Analysis of Variance (ANOVA) for multiple groups comparison. Data fitting a parametric distribution were tested for significance using analysis of Student’s *t*-test and one-way analysis of variance (ANOVA). Non-normally distributed data were analyzed by non-parametric Mann–Whitney *U* test. Significance was defined as *P* < 0.05.

## Results

### Combination of Dexmedetomidine and Netrin-1 Reduced the Neuronal Injury After MCAO Injury

**FIGURE 2:**
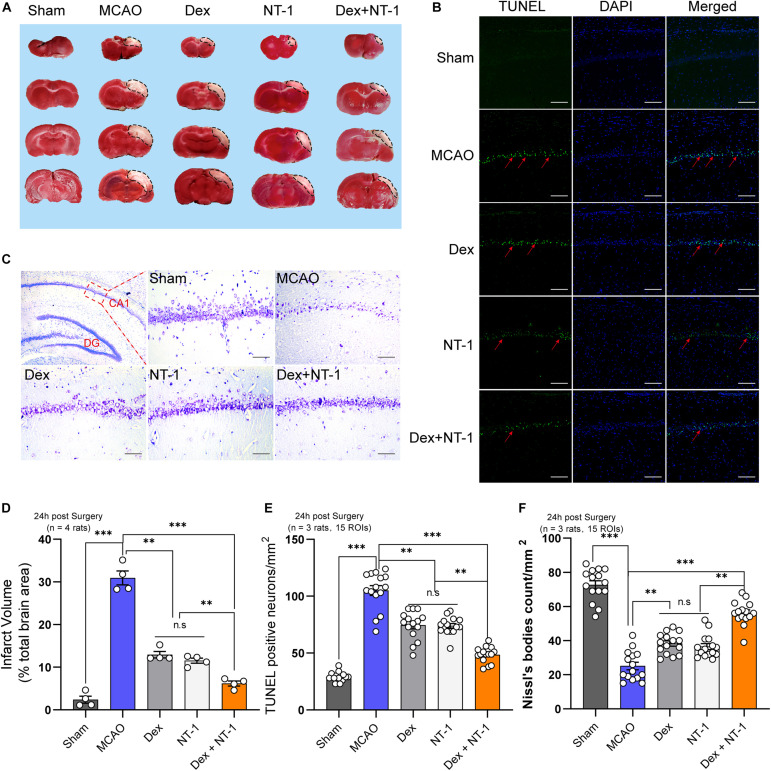
Combination of dexmedetomidine and Netrin-1 reduced the neuronal injury after MCAO injury. **(A)** TTC staining after MCAO injury, the red area represents normal tissue and the white area represents infarcted tissue. **(B)** Representative images of TUNEL-positive cells (green) in the hippocampal CA1 region. The nuclei were stained with DAPI. Scale bar = 200 μm. **(C)** Nissl staining of the surviving cells in the hippocampal CA1 region. Scale bar = 200 μm. **(D)** Percentage of infarct volume after TTC staining. **(E)** Quantitative analysis of TUNEL-positive neurons. **(F)** Quantitative analysis of Nissl-positive neurons. Data are shown as the mean ± SD. ^∗∗^*P* < 0.01 and ^∗∗∗^*P* < 0.001 using one-way ANOVA followed by Tukey’s *post hoc* tests.

TTC staining showed the areas of cerebral infarction. The pale area of brain slices exhibited area of ischemic necrosis (Figures 2A,D). Compared with the Sham group, the infarct volume in MCAO group was distinctly enlarged (*P* < 0.01). After treatment with dexmedetomidine or Netrin-1 alone, the infarct volume was significantly smaller (*P* < 0.01), however, there was no statistical difference between the Dex group and the NT-1 group (*P* > 0.05). When dexmedetomidine was combined with Netrin-1, the cerebral infarction volume was further reduced (*P* < 0.01).

Next, we further analyzed neuronal apoptosis and necrosis in the CA1 region of hippocampus. The numbers of TUNEL-positive cells in the MCAO group were higher than those in the sham group (*P* < 0.01). Both dexmedetomidine and Netrin-1 treatment significantly reduced apoptosis (*P* < 0.01), but no significant difference existed among the Dex and NT-1 group (*P* > 0.05). However, combined with the dexmedetomidine and Netrin-1, the numbers of TUNEL-positive cells significantly decreased (*P* < 0.01) (Figures 2B,E).

Nissl staining showed the number of neurons alive in the CA1 region of hippocampus (Figure 2C). Meanwhile, Nissl’s bodies were showed in the cytoplasm. Treatment with dexmedetomidine or Netrin-1 increased the numbers of Nissl’s bodies at 24 h after reperfusion as compared with the MCAO group (*P* < 0.01). However, the effect not as effectively as combination treatment (Dex + NT-1 group) (*P* < 0.01).

### Combination of Dexmedetomidine and Netrin-1 Significantly Promoted the Recovery of Neurological Function After MCAO Injury

**FIGURE 3:**
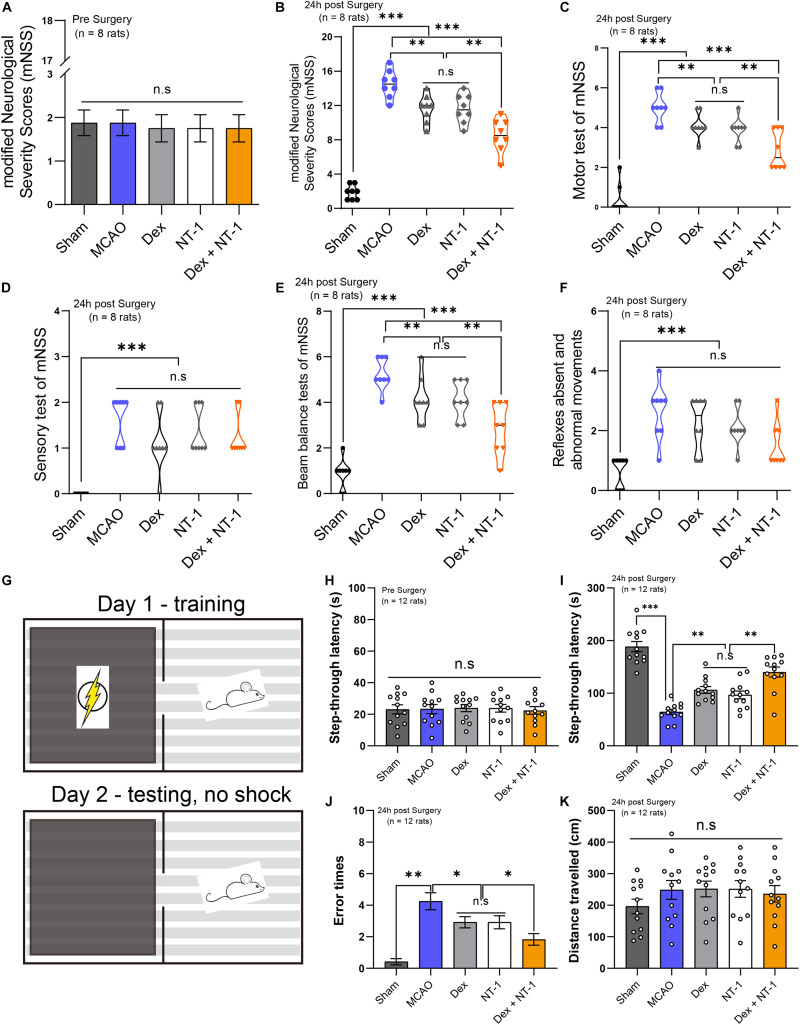
Combination of dexmedetomidine and Netrin-1 promoted the recovery of neurological function and alleviated the cognitive impairment after MCAO injury. **(A)** The basic values of modified Neurological Severity Scores (mNSS) of all experimental rats before MCAO model. **(B)** The mNSS of all experimental rats at 24 h after MCAO model. **(C)** The Motor test of mNSS. **(D)** The Sensory test of mNSS. **(E)** The Beam balance test of mNSS. **(F)** Reflexes absent and abnormal movements test of mNSS. Data are shown as the mean ± SD, *n* = 8. **P < 0.01 and ***P < 0.001 using one-way ANOVA followed by Tukey’s *post hoc* tests. **(G)** The schematic diagram of the step-through test. **(H)** The basic values of step-through latency of all experimental rats before MCAO model. **(I)** The latency to enter into the dark room of all experimental rats at 24 h after MCAO model. **(J)** The error times to enter into the dark room. **(K)** The total distance traveled. Data are shown as the mean ± SD, *n* = 12. ^∗^*P* < 0.05, ^∗∗^*P* < 0.01, ^∗∗∗^*P* < 0.001 using one-way ANOVA followed by Tukey’s *post hoc* tests.

First of all, before the establishment of MCAO model, we measured the basic values of modified Neurological Severity Scores (mNSS) of all experimental rats. The results showed that there was no statistical difference in mNSS scores between all groups (*P* > 0.05) (Figure 3A). mNSS were significantly higher in the MCAO group than in the sham group 24 h after reperfusion (*P* < 0.01). Treatment with dexmedetomidine or Netrin-1 decreased the mNSS as compared to the MCAO group (*P* < 0.01). When dexmedetomidine was combined with Netrin-1, the mNSS was further decreased (*P* < 0.01) (Figure 3B).

We made separate statistics for the different items (Motor test, Sensory test, Beam Balances test, Reflexes absent, and abnormal movements) of mNSS (Figures 3C–F). In the Motor test and Beam Balances test, the score of Dex + NT-1 group was significantly lower than that of the single drug group (*P* < 0.01) (Figures 3C,E). However, for the Sensory test and the Reflexes absent and abnormal movements, there was no significant difference among the scores in the MCAO, Dex, NT-1, and Dex + NT-1 groups (*P* > 0.05) (Figures 3D,F).

### Combination of Dexmedetomidine and Netrin-1 Significantly Alleviated the Cognitive Impairment After MCAO Injury

First of all, before the establishment of MCAO model, we measured the basic values of step-through latency of all experimental rats. The results showed that there was no statistical difference in step-through latency between all groups (*P* > 0.05) (Figure 3H). After MCAO at 24 h, the MCAO group displayed a reduced latency to enter into the dark room compared with the Sham group (*P* < 0.01). Treatment with dexmedetomidine or Netrin-1 prolonged the step-through latency as compared to the MCAO group (*P* < 0.01). When dexmedetomidine was combined with Netrin-1, the step-through latency was further prolonged (*P* < 0.01) (Figure 3I). Furthermore, the rats in MCAO group made more error trials in entering the dark room compared with the rats in sham group (*P* < 0.01). However, after the combination of dexmedetomidine and Netrin-1 treatment, the error times were significantly reduced (*P* < 0.01) (Figure 3J). There was no statistical difference in step-through latency and error times between Dex group and NT-1 group (*P* > 0.05).

### Combination of Dexmedetomidine and Netrin-1 Reduced the Neuronal Apoptosis After OGD Injury

**FIGURE 4:**
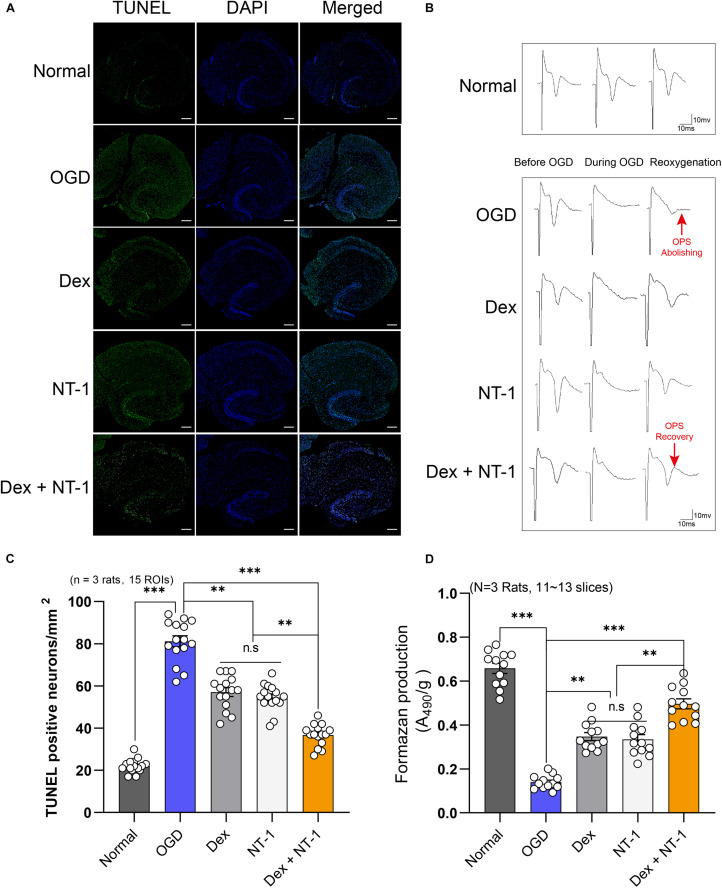
Combination of dexmedetomidine and Netrin-1 reduced the neuronal injury after OGD model. **(A)** Representative images of TUNEL-positive cells (green) in the hippocampal CA1 region. The nuclei were stained with DAPI. Scale bar = 200 μm. **(B)** OPS recordings of the CA1 region of hippocampal slices after reoxygenation treated with drugs. **(C)** Quantitative analysis of TUNEL-positive neurons. **(D)** Formazan production (A490/g) after OGD insult treated with drugs. Data are shown as the mean ± SD. ^∗^*P* < 0.05, ^∗∗^*P* < 0.01 and ^∗∗∗^*P* < 0.001 using one-way ANOVA followed by Tukey’s *post hoc* tests.

*In vivo*, TUNEL-positive cells in the hippocampal CA1 region were analyzed, and OGD experiment was performed for *in vitro* hippocampal slice perfusion, and the changes of TUNEL-positive cells were further analyzed (Figures 4A,C). The numbers of TUNEL-positive cells in the OGD group were higher than those in the Normal group (*P* < 0.01). Both dexmedetomidine and Netrin-1 perfusion treatment reduced apoptosis (*P* < 0.01), but no significant difference existed among the Dex and NT-1 group (*P* > 0.05). However, combined with the dexmedetomidine and Netrin-1, the numbers of TUNEL-positive cells significantly decreased (*P* < 0.01) (Figure 4C).

### Combination of Dexmedetomidine and Netrin-1 in the Treatment of OGD Damaged Hippocampal Slices Can Increase the Formazan Production and Reduce Neuronal Damage

Based on TTC staining, formazan generation was remarkably reduced in the OGD group vs. Normal group. The formazan production of the Dex group and the NT-1 group was significantly higher than that of the OGD group (*P* < 0.01), but no significant difference existed among the Dex and NT-1 group (*P* > 0.05). However, combined with the dexmedetomidine and Netrin-1, the formazan production significantly increased (*P* < 0.01) (Figure 4D).

### Electrophysiology Analysis of OGD Injury and Combination of Dexmedetomidine and Netrin-1 Treatment

The OPS results were showed as described in Figure 4B and Table 1. In OGD group, the OPS abolishing time was 191.92 ± 23.62 s, and the OPS recovery amplitude and rate were all 0%. After dexmedetomidine or Netrin-1 or combination treatment, the abolishing time, recovery amplitude, and rate of OPS were all apparently increased. (OPS abolishing time: 277.16 ± 45.98 s of Dex group, 266.44 ± 48.70 s of NT-1 group, 347.86 ± 51.87 s of Dex + NT-1 group vs. 191.92 ± 23.62 s of OGD group, *P* < 0.01; OPS recovery amplitude: 40.79 ± 8.35% of Dex group, 39.41 ± 8.61% of NT-1 group, 59.37 ± 6.56% of Dex + NT-1 group vs. 0% of OGD group, *P* < 0.01; OPS recovery rate: 75% of Dex + NT-1 group vs. 0% of OGD group, *P* < 0.01).

**TABLE 1:** OPS recordings of the CA1 region of hippocampal slices after reoxygenation treated with drugs.

Groups	*N* (Rats)	*n* (Slices)	OPS abolishing time (s)	OPS recovery time (s)	OPS recovery amplitude (%)	OPS recovery rate (%)
OGD	3	13	191.92 ± 23.62	–	0	0(0/13)
Dex	3	12	277.16 ± 45.98*#	682.72 ± 93.30^#^	40.79 ± 8.35*#	25.00(3/12)*#
NT-1	3	12	266.44 ± 48.70*#	680.38 ± 111.40^#^	39.41 ± 8.61*#	33.33(4/12)*#
Dex + NT-1	3	12	347.86 ± 51.87*	587.73 ± 98.58	59.37 ± 6.56*	75(9/12)*

Results were presented as mean ± SD, N = 3 rats, n = 11 ∼ 13 Hippocampal slices. *P < 0.01 vs. OGD group; ^#^P < 0.01 vs. Dex + NT-1 group.

### Effects of Dexmedetomidine and Netrin-1 on ERS Protein (CHOP and GRP78) Expression After MCAO and OGD Injury

**FIGURE 5:**
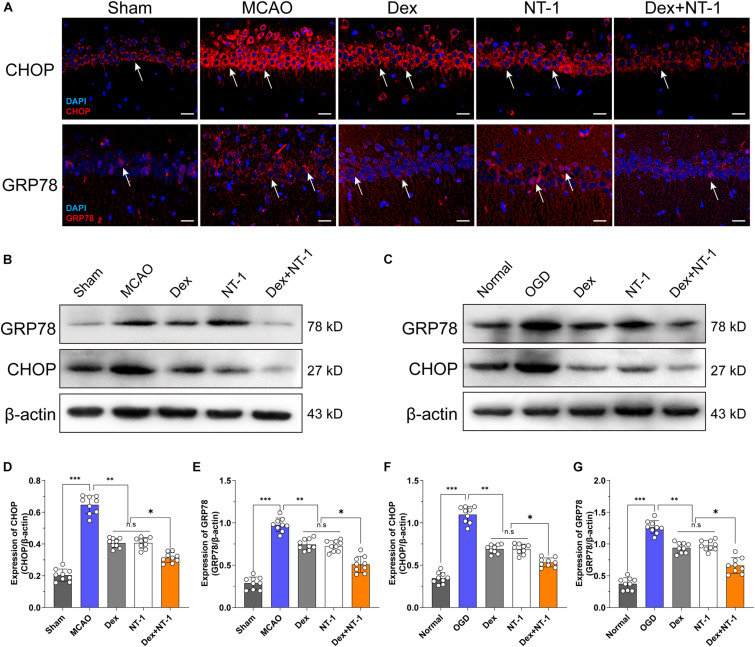
Effects of dexmedetomidine and Netrin-1 on ERS protein (CHOP and GRP78) expression after MCAO and OGD injury. **(A)** CHOP (first line) and GRP78 (second line) expressions in hippocampus after MCAO injury. Immunofluorescence staining exhibited positive cells of CHOP and GRP78. Red indicated CHOP and GRP78 expression in plasm of pyramidal neurons, and blue indicated the nucleus. Scale bar = 50 μm. **(B,C)** Western blot analysis of ERS-related proteins CHOP and GRP78, β-actin was used as an internal control. **(D–G)** Quantitative analysis of CHOP and GRP78 expression levels. ^∗^*P* < 0.05, ^∗∗^*P* < 0.01 and ^∗∗∗^*P* < 0.001 using one-way ANOVA followed by Tukey’s *post hoc* tests.

Immunofluorescence and Western blot analyses demonstrated weak, cytoplasmic CHOP and GRP78 expression in the hippocampal tissues in the Sham group (Figure 5A). Compared with the Sham group, the expression of CHOP and GRP78 gradually increased after MCAO and OGD injury (*P* < 0.01), but decreased in the Dex, NT-1, and Dex + NT-1 groups (*P* < 0.01), the inhibition of CHOP and GRP78 protein expression was more obvious in the Dex + NT-1 group (*P* < 0.01). However, there was no significant difference existed among the Dex and NT-1 group (*P* > 0.05) (Figure 5).

### Dexmedetomidine and Netrin-1 Enhanced the Activation of the ERK5/MEF2A Signaling Pathway After MCAO Injury and OGD Injury

**FIGURE 6:**
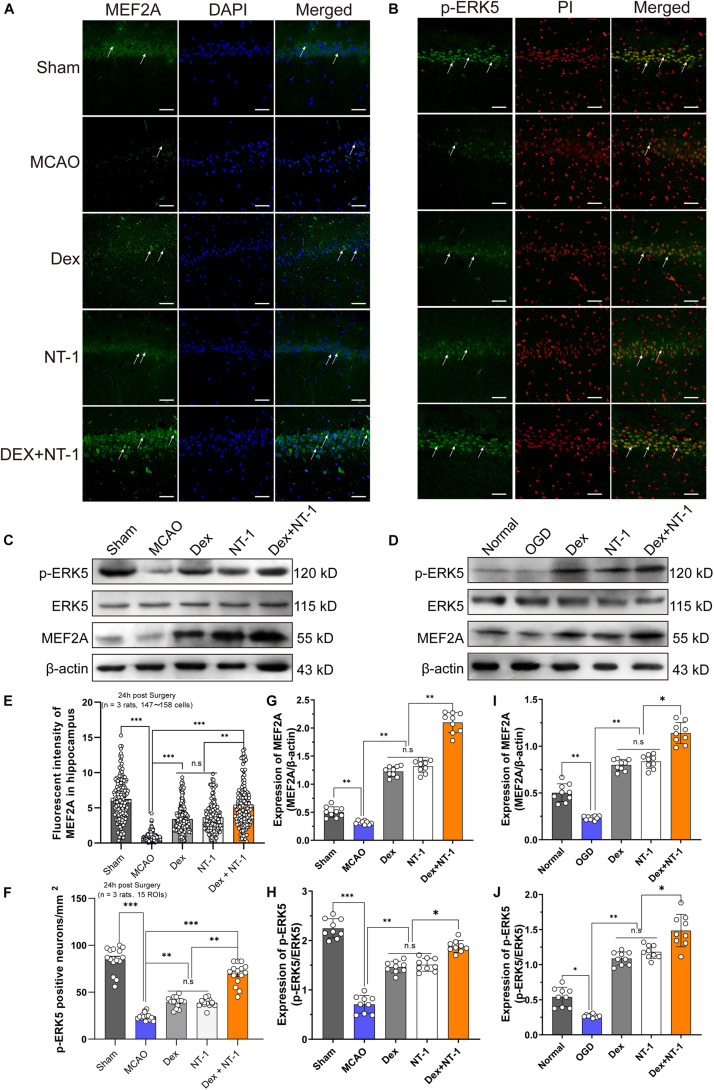
Dexmedetomidine and Netrin-1 up-regulated the expression of the ERK5 and MEF2A after MCAO injury and OGD injury. **(A)** MEF2A expressions in hippocampus after MCAO injury. Immunofluorescence staining exhibited positive cells of MEF2A. Green indicated MEF2A expression in plasm of pyramidal neurons, and blue indicated the nucleus. Scale bar = 100 μm. **(B)** p-ERK5 expressions in hippocampus after MCAO injury. Immunofluorescence staining exhibited positive cells of p-ERK5. Green indicated p-ERK5 expression in nucleus of pyramidal neurons, and red indicated the nucleus. Scale bar = 100 μm. **(C,D)** Western blot analysis of MEF2A, ERK5 and p-ERK5 proteins, β-actin was used as an internal control. **(E–I)** Quantitative analysis of MEF2A and GRP78 expression levels. **(F–J)** Quantitative analysis of p-ERK5 expression levels. ^∗^*P* < 0.05, ^∗∗^*P* < 0.01 and ^∗∗∗^*P* < 0.001 using one-way ANOVA followed by Tukey’s *post hoc* tests.

To further confirm the expression level of ERK5/MEF2A, we utilized immunofluorescence staining. Green fluorescence staining represented the target protein, and red (PI) or blue (DAPI) fluorescence staining represented the cell nucleus (Figures 6A,B). Immunofluorescence showed that the MEF2A was mainly located in cytoplasm (Figure 6A) and p-ERK5 was mainly expressed in nucleus (Figure 6B). Immunofluorescence and Western blot analyses demonstrated weak MEF2A and p-ERK5 expression in the hippocampal tissues after MCAO and OGD injury. Both in MCAO and OGD models, Dexmedetomidine and Netrin-1 significantly increased MEF2A expression and promoted ERK5 phosphorylation (*P* < 0.01), especially in the Dex + NT-1 group, the protein activation level was more obvious (*P* < 0.01), whereas total ERK5 did not change after MCAO and OGD injury in all groups (*P* > 0.05) (Figures 6C–J).

### Knockdown MEF2A Significantly Weakened the Therapeutic Effect of Dexmedetomidine Combined With Netrin-1 After MCAO and OGD Injuries

**FIGURE 7:**
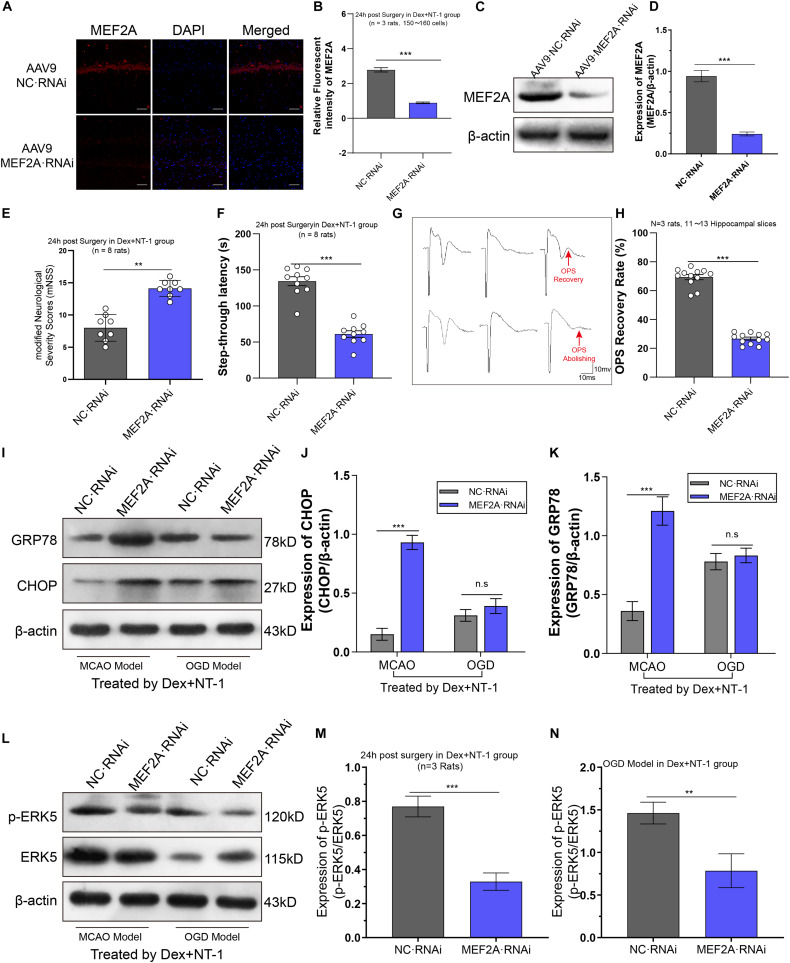
Knockdown MEF2A weakened the therapeutic effect of dexmedetomidine combined with Netrin-1 after MCAO and OGD injuries. **(A,B)** Immunofluorescence staining exhibited downregulation in MEF2A expression in the hippocampus at 14 days after AAV9-MEF2A RNAi microinjection. **(C,D)** Western blot analysis shows downregulation in MEF2A expression in the hippocampus at 14 days after AAV9-MEF2A RNAi microinjection. **(E)** The mNSS result after MEF2A knockdown. **(F)** The step-through latency result after MEF2A knockdown. **(G,H)** The OPS recovery rate after MEF2A knockdown. **(I–K)** Western blot analysis of ERS-related proteins CHOP and GRP78 in the hippocampus at 14 days after AAV9-MEF2A RNAi microinjection, β-actin was used as an internal control. **(L–N)** Western blot analysis of ERS-related proteins ERK5 and p-ERK5 in the hippocampus at 14 days after AAV9-MEF2A RNAi microinjection, β-actin was used as an internal control. ^∗∗^*P* < 0.01 and ^∗∗∗^*P* < 0.001 using one-way ANOVA followed by Tukey’s *post hoc* tests.

To further verify that the therapy effect of dexmedetomidine combined with Netrin-1 was activated the transcription of MEF2A, *In vivo* knockdown of MEF2A was performed by stereotactic injection of AAV9-MEF2A RNAi into the ischemic hippocampus. First, we assessed MEF2A silencing efficiency by immunofluorescence and western blot to analyze MEF2A protein levels in the hippocampus. We found that AAV9-MEF2A RNAi rats exhibited reduced MEF2A protein expression compared with AAV9-NC RNAi rats at 15 days after injection (*P* < 0.01) (Figures 7A–D).

In MCAO model, we observed the effect of dexmedetomidine combined with Netrin-1 in improving neurological function was significantly eliminated by pre-knockdown MEF2A (Figures 7E,F). Compared with AAV9-NC RNAi group, the mNSS increased and the step-through latency decreased significantly in AAV9-MEF2A RNAi group (*P* < 0.01).

In OGD model, we observed the effect of dexmedetomidine combined with Netrin-1 in improving OPS recovery amplitude and rate was significantly eliminated by pre-knockdown MEF2A (Figures 7G,H). The OPS recovery rate in AAV9-MEF2A RNAi group was significantly lower than AAV9-NC RNAi group (*P* < 0.01).

### Knockdown MEF2A Significantly Inhibited Effect of Dexmedetomidine Combined With Netrin-1 on the Expression of ERS Proteins (CHOP and GRP78)

Through western blot analysis (Figures 7I–K), in MCAO model, compared with AAV9-NC RNAi group, the expression of CHOP and GRP78 were significantly higher than AAV9-MEF2A RNAi group (*P* < 0.01). Whereas in the OGD model, CHOP and GRP78 showed no statistically significant difference between AAV9-NC RNAi group and AAV9-MEF2A RNAi group (*P* > 0.05).

### Knockdown of MEF2A Inhibited the Phosphorylation of ERK5 by Dexmedetomidine Combined With Netrin-1 Treatment

To further analyze the effect of knockdown MEF2A on ERK5 phosphorylation, we first injected AAV9-MEF2A RNAi, then we established MCAO injury and OGD injury models, and administered dexmedetomidine and Netrin-1 combined intervention. Western blot was used to detect the expression of ERK5 and its phosphorylated proteins (Figure 7L). We found that both in the MCAO model and the OGD model, pre-knockdown MEF2A significantly reduced phosphorylation levels of ERK5 compared with the AAV9-NC RNAi group (*P* < 0.01), while the total ERK5 expression levels were almost unaffected (*P* > 0.05) (Figures 7M,N).

## Discussion

Perioperative stroke seriously affects the prognosis of patients, so we urgently need to find effective prevention and treatment measures. It has been reported that dexmedetomidine plays an important role in perioperative brain protection (Ji et al., 2013). Moreover, some studies have confirmed that dexmedetomidine displayed more advantages, including Inhibition of inflammation, apoptosis resistance and edema reduction (Zhai et al., 2019; Zhu et al., 2019), but the protective effect of dexmedetomidine is usually limited to the early stage of cerebral ischemia, and we expect to seek a drug that can enhance the effect of dexmedetomidine. Previous studies have found that Netrin-1 has a protective effect on the permeability of the blood-brain barrier and can stabilize neurons without sustained damage after cerebral ischemia, which is related to the alleviation of apoptosis (Wu et al., 2008). Thus, in view of the protective advantages, this study was formulated to prove the better synergistic function of combination therapy.

Based on the results of this study, both *in vivo* and *in vitro* experiments showed that cerebral ischemia and hypoxia would cause significant neuron damage, MCAO model rats showed significant neurological dysfunction and cognitive impairment, and electrophysiological tests showed a significant decrease in OPS recovery. After the intervention of dexmedetomidine and Netrin-1, the injury could be alleviated, which was mainly manifested as: TTC staining showed reduced infarction volume, decreased TUNEL apoptotic neurons, increased neuronal Nissl’s bodies, decreased mNSS, and increased OPS recovery.

Based on the data before the experiment and previous research, we selected dexmedetomidine (9 μg/kg) and Netrin (500 ng) (Kuhmonen et al., 2001; Yu et al., 2017). In neurobehavioral experiments, we found that combined treatment can reduce the total score of mNSS. We analyzed its modules and found that dexmedetomidine combined with Netrin has a stronger effect in improving motor function. Compared with single-drug therapy, there was no statistical difference between epilepsy and other reflex or sensory treatments. The reason may be that dexmedetomidine has sedative and anti-epileptic effects (Mirski et al., 1994; Oda et al., 2007). Unexpectedly, the combination therapy of dexmedetomidine and Netrin-1 showed a protective effect superimposed on the effect of a single drug. The superimposed effect of this protection phenomenon is worthy of further study.

Cerebral ischemia-reperfusion injury can trigger endoplasmic reticulum stress to induce neuronal apoptosis. CHOP and GRP78 are the key molecules of endoplasmic reticulum stress. This study found that after 24 h of cerebral ischemia-reperfusion injury, immunofluorescence staining and western blot analysis showed that the expression of CHOP and GRP78 was significantly up-regulated. Dexmedetomidine, Netrin, and the combination treatment group significantly down-regulated the expression of key proteins of endoplasmic reticulum stress, and this change is consistent with the trend of behavioral phenotype and morphology. This result suggests that the two drugs can reduce neuronal damage by inhibiting the endoplasmic reticulum stress protein to exert anti-apoptotic effects. In order to further explore, we established the OGD model and found the same results *in vitro*. Dexmedetomidine and Netrin perfused isolated hippocampal slices can also down-regulate the expression of CHOP and GRP78, and the down-regulation effect of combined treatment was more obvious. It was consistent with the trend of electrophysiological results. Brain tissue is damaged due to ischemia and hypoxia, which increases the influx of Ca^2+^ in the cell, leading to Ca^2+^ overload. Ca^2+^ overload leads to the loss of endoplasmic reticulum Ca^2+^ homeostasis, and Ca^2+^ is released from the endoplasmic reticulum into the cytoplasm, leading to Ca^2+^ dependent protease dysfunction (Gissel et al., 1997; Yu et al., 2018). Finally, unfolded, or misfolded protein precursors gather in the endoplasmic reticulum, triggering the endoplasmic reticulum stress process.

Studies have found that a variety of stimulating factors, such as inflammatory factors, high osmotic pressure, hypoxia, etc., can activate the ERK5 signaling pathway, translocate ERK5 activation from the cytoplasm to the nucleus, and then exert anti-inflammatory and anti-apoptotic effects. After cerebral ischemia-reperfusion injury, the expression of phosphorylated ERK5 (p-ERK5) can be increased by activating the ERK5 signaling pathway, thereby inhibiting the transformation of Bad to a pro-apoptotic form, and playing an anti-apoptotic effect on neurons (Wang et al., 2006; Gu et al., 2017). ERK5 can activate Myocyte enhancer-binding factor 2 (MEF2) to give it transcriptional properties (Pang et al., 2020). This study found that after MCAO and OGD injury, the expression of MRF2A and the phosphorylation level of ERK5 decreased significantly. After the intervention of dexmedetomidine and Netrin, the expression of MEF2A and the phosphorylation level of ERK5 can be significantly reversed and up-regulated. The number of damaged neurons was significantly reduced compared with the damage, suggesting that Netrin-1 and dexmedetomidine, as neurotrophic factors, can activate the ERK5-MEF2A signaling pathway, and play a neuroprotective effect by up-regulating the expression of pERK5 and MEF2A.

In order to further verify that dexmedetomidine and Netrin-1 inhibit the endoplasmic reticulum stress by regulating the phosphorylation of ERK5 to regulate the transcriptional activity of MEF2A, we used an adeno-associated virus vector and stereotaxic microinjection to inhibit MEF expression. Interestingly, knockdown MEF2A, the therapeutic effect of dexmedetomidine combined with Netrin is significantly weakened, the neurological damage in rats is aggravated, the cognitive function is impaired, and the expression of CHOP and GRP78 was significantly up-regulated. The OPS recovery after OGD injury was significantly weakened, but there was no significant difference in the expression of CHOP and GRP78. It may be that knockdown MEF2A may block the neuroendocrine function of the isolated brain slice, or it may be due to the shorter time *in vitro* experiment, not enough to cause changes in the expression of key proteins.

The above experimental results suggest that MEF2A serves as a key regulatory target for dexmedetomidine and Netrin to exert neuroprotective effects. To explore the relationship between ERK5 and MEF2A, we analyzed the phosphorylation level of ERK5 in hippocampus of MCAO model and OGD model by western blot. The results showed that pre-knockdown of MEF2A, the phosphorylation level of ERK5 after MCAO injury or OGD injury was significantly reduced, which suggests that MEF2A can promote the phosphorylation of ERK5 to play a transcriptional regulation process and affect the neuronal endoplasmic reticulum stress pathway.

There are still some limitations in the current work. The potential pharmacokinetic interaction between dexmedetomidine and Netrin is unclear. Our current study cannot directly prove that these two drugs can directly inhibit ER stress proteins, we only found that drugs may indirectly regulate CHOP and GRP78 through ERK5/MEF2A pathway, and our hypothesis can be further verified by subsequent experiments including knocking-down of CHOP. In addition, the method of administration of Netrin is lateral ventricle microinjection. Whether it can successfully penetrate the blood-brain barrier is still unclear, so further research is needed to promote the clinical treatment of Netrin.

In summary, this study demonstrates the neuroprotective effects of dexmedetomidine combined with Netrin on cerebral ischemia-reperfusion injury and hippocampal hypoxia injury in terms of ERS, apoptosis, electrophysiology, etc. The synergistic effect of combination therapy is related to the activation of ERK5/MEF2A signaling pathway. Collectively, our results provided valid evidence on the treatment of perioperative stroke.

## Data Availability Statement

The raw data supporting the conclusions of this article will be made available by the authors, without undue reservation.

## Ethics Statement

All animal experiments were conducted in accordance with the National Institute of Health Guide for the Care and Use of Laboratory Animals. The experimental procedures were approved by the Animal Research Committee by Shihezi University (protocol A2018-025-01) and West China Hospital of Sichuan University (protocol 2018159A).

## Author Contributions

Y-XZ, SW, and J-WY contributed to the study design and revised the manuscript. J-WY, JL, and R-XW conducted the experiment and collected and analyzed the data. Y-MR and YL provided assistance in experiment performing. J-WY and JL wrote the manuscript. All authors gave final approval of the manuscript.

## Conflict of Interest

The authors declare that the research was conducted in the absence of any commercial or financial relationships that could be construed as a potential conflict of interest.
